# BCOR Biology in Development and Cancer

**DOI:** 10.3390/cells15201856

**Published:** 2026-10-13

**Authors:** Hitomi N. Royston, Alberto Taddia, Fabio R. Santori, Natalia Ivanova, Kosuke Funato

**Affiliations:** 1 Center for Molecular Medicine, University of Georgia, Athens, GA 30602, USA; hitomi.nakasato@uga.edu (H.N.R.); fabio.santori@uga.edu (F.R.S.);; 2 Department of Biochemistry and Molecular Biology, University of Georgia, Athens, GA 30602, USA

**Keywords:** BCOR, BCOR-ITD, PRC1.1, Polycomb regulation, chromatin regulation, development, cancer

## Abstract

BCOR is a transcriptional corepressor and a core component of Polycomb repressive complex 1.1 (PRC1.1) with diverse roles in development and disease. Germline loss-of-function *BCOR* mutations cause oculofaciocardiodental syndrome, whereas diverse somatic alterations drive various malignancies. In myeloid neoplasms, loss-of-function truncating mutations disrupt BCOR-mediated transcriptional repression and cooperate with concurrent oncogenic events. Meanwhile, *BCOR* fusions and internal tandem duplications define distinct tumor entities in sarcomas and brain tumors. Growing evidence indicates that these somatic alterations are not mechanistically equivalent and that the functional impact of *BCOR* alterations is highly context-dependent, varying across cell types and developmental stages. How these distinct mutations disrupt molecular programs to drive oncogenesis remains a central question in the field. This review summarizes the current knowledge of BCOR domain organization, its epigenetic and physiological functions, and the emerging mutational landscape in human malignancies.

## 1. Introduction

BCOR (BCL6 corepressor) was originally identified as a transcriptional corepressor and later recognized as a core component of non-canonical Polycomb repressive complex 1.1 (PRC1.1) [1,2]. BCOR functions as a scaffold that tethers sequence-specific DNA-binding factors to chromatin-modifying machinery [1]. Within PRC1.1, BCOR coordinates chromatin modification and transcriptional repression across a broad range of Polycomb target loci [2,3,4,5].

BCOR is essential for normal development; germline *BCOR* mutations cause oculofaciocardiodental (OFCD) syndrome in humans [6]. Studies across multiple model systems have further demonstrated that BCOR regulates key developmental processes, including early embryogenesis, lineage specification, and tissue-specific differentiation [6,7,8,9,10,11,12].

Advances in genomic profiling have revealed recurrent *BCOR* alterations across a wide range of cancers, including hematologic malignancies, sarcomas, and central nervous system (CNS) tumors [13,14,15,16]. These alterations include loss-of-function mutations, gene fusions, and internal tandem duplications (ITDs), each exerting distinct biological effects. This heterogeneity complicates the classification of *BCOR* as strictly a tumor suppressor gene or an oncogene. Instead, its role in oncogenesis is highly context-dependent, governed by both the specific type of genetic alteration and the cellular or tissue lineage [13,14,17].

This review outlines the history and major advances of BCOR research. We detail its molecular framework, epigenetic functions, developmental roles, and involvement in human disease. Finally, we examine the spectrum of oncogenic *BCOR* alterations, focusing specifically on CNS tumors harboring BCOR-ITD. By integrating insights from developmental biology and cancer genomics, we highlight the context-dependent nature of BCOR function and discuss why lineage-relevant models are essential to decipher its role in oncogenesis.

## 2. History of BCOR Research

BCOR was originally identified in 2000 as a binding partner of the POZ/BTB domain of BCL6, a transcriptional repressor critical for germinal center B-cell development and lymphomagenesis [18,19]. BCOR enhances BCL6-mediated transcriptional repression and associates with both class I and class II histone deacetylases (HDACs) [1]. Although initially characterized for its interaction with BCL6, subsequent studies revealed that BCOR function extends beyond BCL6-dependent repression. Yeast two-hybrid and subsequent reporter assays showed that specific BCOR isoforms interact with AF9 to suppress AF9-mediated transcriptional activity [20]. BCOR is also a component of the FBI-1 complex, where it is proposed to repress the cell-cycle regulators *RB* and *CDKN1A* [21,22]. Additionally, BCOR represses the growth factor *EREG* during mesenchymal differentiation through its interaction with FBXL11 (KDM2A) [23].

The developmental importance of BCOR became clear in 2004, when germline *BCOR* mutations were identified as the genetic cause of oculofaciocardiodental (OFCD) syndrome in humans [6]. These findings were subsequently validated across diverse model systems. In mice, *Bcor* is broadly expressed during embryogenesis and is required for early lineage specification and extraembryonic development [8,9]. Concurrently, studies in *Xenopus tropicalis* demonstrated that Bcor is indispensable for normal left–right patterning. Knockdown of *Bcor* induced reversed cardiac orientation and abnormal gut patterning [7]. Furthermore, investigations using human mesenchymal stem cells derived from OFCD patients confirmed that BCOR modulates both osteogenic and dentinogenic differentiation [10].

A major breakthrough occurred in 2006 when BCOR was identified as a core component of the chromatin-modifying Polycomb group (PcG) complex [2]. This discovery expanded the understanding of BCOR beyond BCL6-targeted repression. Subsequent work linked BCOR-associated Polycomb activity to CpG-island targeting and monoubiquitination of histone H2A at lysine 119 (H2AK119ub1) [3,4]. This multi-protein assembly was later classified as PRC1.1 [5]. X-ray crystallography revealed the binding interface between the BCOR PCGF Ub-like fold discriminator (PUFD) domain and the PCGF1 RING finger- and WD40-associated ubiquitin-like (RAWUL) domain [24]. Functional studies in human embryonic stem cells (hESCs) demonstrated that BCOR-associated PRC1.1 is essential for maintaining a primed pluripotent state by actively repressing genes associated with mesodermal and endodermal differentiation [11].

Beginning in the early 2010s, high-throughput genomic profiling identified *BCOR* as a recurrently altered gene across several malignancies. Somatic loss-of-function *BCOR* mutations were first characterized in acute myeloid leukemia (AML), suggesting a tumor-suppressor role in myeloid malignancies [13]. Subsequent studies extended these findings to myelodysplastic syndromes (MDSs) and related disorders, confirming the widespread distribution of *BCOR* alterations in myeloid malignancies [25]. Simultaneously, recurrent genomic alterations involving *BCOR* were detected in pediatric and mesenchymal tumors. These include *BCOR::CCNB3* fusions in undifferentiated small round-cell sarcomas [14] as well as BCOR-ITD in clear-cell sarcoma of the kidney (CCSK) [15], undifferentiated round-cell sarcomas (URCSs) of infancy, and primitive myxoid mesenchymal tumors of infancy (PMMTI) [26]. DNA methylation profiling further identified high-grade neuroepithelial tumors with *BCOR* alteration as a molecularly distinct entity among pediatric brain tumors [16].

By the 2020s, the focus of BCOR research shifted from genomic discovery toward defining the biological, diagnostic, and clinical implications of these alterations. This evolution is reflected in the updated World Health Organization (WHO) classifications. In the 2020 WHO classification of soft-tissue and bone tumors, sarcomas with *BCOR* genetic alterations were codified within the category of undifferentiated small round-cell sarcomas of bone and soft tissue [27]. The 2021 WHO classification of CNS tumors subsequently recognized “CNS tumor with BCOR internal tandem duplication” as a distinct embryonal tumor subtype [28]. Although BCOR-ITD mutations occur in both intracranial brain tumors and extracranial sarcomas, comparative studies indicate they represent distinct entities rather than the same disease arising at different anatomical locations [17]. DNA methylation profiling further showed that soft-tissue and kidney tumors cluster separately from CNS tumors despite sharing *BCOR* alterations [29].

## 3. Molecular Organization and Functions of BCOR

### 3.1. Gene, Isoforms, and Paralog of BCOR

The human BCL6 corepressor (*BCOR*) gene is located on the X chromosome at cytoband Xp11.4 [30]. It typically consists of 15 exons, with the principal canonical transcript (ENST00000378444) encoding a 1755-amino-acid nuclear protein with an estimated molecular mass of 192 kDa (Figure 1A). BCOR is ubiquitously expressed across mammalian tissues in both human and mouse, a feature consistent with its fundamental roles in transcriptional regulation, hematopoiesis, and development [1,6,8,9,30].

*BCOR* pre-mRNA undergoes extensive alternative splicing to generate multiple transcript variants and protein isoforms [8,30]. These isoform-specific sequence differences can alter BCOR function by retaining or excluding regions involved in protein–protein interactions. For instance, AF9 (also known as MLLT3) selectively binds only to BCOR isoforms that retain a specific 34-amino-acid internal sequence [20]. In transcriptional reporter assays, BCOR isoforms a and b (which retain the AF9-binding domain) suppressed AF9-mediated transcriptional activation, whereas isoform c (which lacks this sequence) failed to exert an inhibitory effect [20]. In another example, alternative RNA processing generated a transcript with a shorter open reading frame that encodes a truncated isoform lacking BCL6-dependent corepressor activity [1]. These findings indicate that isoform-specific alternative splicing changes BCOR-binding partners and downstream gene regulation.

BCOR-like 1 (*BCORL1*) is a paralog of *BCOR* [31]. The *BCORL1* gene maps to a distinct locus on the X chromosome at Xq26.1 [32]. The principal transcript of *BCORL1*, ENST00000540052.6 (*BCORL1*-204), contains 14 exons and encodes a nuclear protein of 1785 amino acids [32] (Figure 1A). BCOR and BCORL1 share conserved C-terminal regions containing ankyrin repeats and the PCGF1-binding PUFD domain [30,31]. Despite these C-terminal similarities, the two paralogs differ significantly across their N-terminal architecture and overall domain topologies. Unlike BCOR, BCORL1 lacks canonical BCL6- and MLLT3-binding domains; instead, it features an N-terminal PXDLS motif that interacts with C-terminal binding protein (CtBP), alongside multiple nuclear receptor-coactivator LXXLL motifs [30,31]. Differences in domain organization likely influence how BCOR and BCORL1 recruit corepressors. Cell-based reporter assays indicate that BCORL1-mediated transcriptional repression depends primarily on CtBP, with class II histone deacetylases contributing in part [31].

Germline alterations in *BCOR* and *BCORL1* are associated with different developmental syndromes. Heterozygous loss-of-function *BCOR* variants cause oculofaciocardiodental (OFCD) syndrome, predominantly in females, whereas selected hemizygous variants are associated with Lenz microphthalmia syndrome and related ocular phenotypes [6,12,30]. Hemizygous *BCORL1* variants are associated with Shukla–Vernon syndrome and related neurodevelopmental phenotypes [33,34]. Somatic mutations in both genes occur in myeloid neoplasms, including AML and MDS, though they present with highly distinct mutational frequencies [13,25,35]. In contrast, *BCOR* gene fusions and ITDs uniquely define specific bone sarcomas, CNS tumors, and clear-cell sarcomas of the kidney [14,15,16]. Taken together, these clinical and molecular findings demonstrate that BCOR and BCORL1 serve different functions across developmental and cancer contexts. Table 1 summarizes the shared and distinct molecular features, interaction partners, and disease associations of BCOR and BCORL1.

**Table 1:** Comparison of BCOR and BCORL1. Abbreviations: aa, amino acid; CtBP, C-terminal-binding protein; HDAC, histone deacetylase; ITD, internal tandem duplication; OFCD, oculofaciocardiodental syndrome; PRC1.1, Polycomb repressive complex 1.1; PUFD, PCGF ubiquitin-like fold discriminator.

Feature	BCOR	BCORL1	Evidence and Limitations	References
Gene locus and protein length	Xp11.4; 1755 aa	Xq26.1; 1785 aa (long isoform)	Sequence annotation	[30,32]
PRC1.1 interface	PUFD binds PCGF1	PUFD binds PCGF1	Structural and biochemical studies	[24,36]
Other interactions	BCL6; isoform-specific AF9/MLLT3	CtBP via the PXDLS motif; class II HDACs	Interaction and reporter assays	[1,20,31]
Paralog relationship	BCOR-containing PRC1.1 complexes	Distinct BCORL1-containing PRC1.1 complexes	Overlapping targets and partial functional compensation in leukemia models	[37]
Developmental phenotypes	Heterozygous loss-of-function variants cause OFCD (X-linked dominant; predominantly affected females); selected hemizygous variants cause Lenz microphthalmia and related ocular phenotypes	Male infertility in mice carrying a 22-bp frameshift deletion in *Bcorl1* exon 3; hemizygous *BCORL1* variants are associated with Shukla–Vernon syndrome and related neurodevelopmental phenotypes	Human genetics and mouse models; *BCORL1*-associated neurodevelopmental evidence is based on small clinical series, and the molecular mechanism remains unresolved	[6,12,34,38,39,40]
Cancer alterations	Loss-of-function variants, ITDs, and fusions	Myeloid loss-of-function variants; rare tumor rearrangements	Tumor sequencing; direct PRC1.1 evidence in leukemia models	[13,14,15,16,25,35,37,41]

### 3.2. Protein Domains and Functional Motifs of BCOR

BCOR is a multidomain nuclear protein that primarily serves as a component of transcriptional repressor complexes [1,2,30]. Its functional architecture comprises several distinct regions: an N-terminal BCL6-binding domain (BBD), central tandem ankyrin repeats, an isoform-specific AF9/MLLT3-interacting motif, and a highly conserved C-terminal PCGF Ub-like fold discriminator (PUFD) domain [1,20,24,30] (Figure 1A).

The N-terminal BBD interacts with the POZ/BTB domain of the transcriptional repressor BCL6 [1]. Binding assays showed that BCOR interacts selectively with BCL6 rather than the other POZ-domain proteins [1]. At this site, BCOR directly competes with other canonical corepressors, such as N-CoR and SMRT, for access to the BCL6 POZ/BTB domain. X-ray crystallography indicates that a BCOR-derived peptide binds within a lateral groove of the BCL6 BTB domain dimer [42]. Beyond its canonical role in interacting with BCL6, the BBD region also interacts with IRF8 to modulate immune and hematopoietic gene networks [43].

The central region of BCOR features tandem ankyrin repeats, which serve as protein interaction domains found in many regulatory proteins [30]. However, the precise mechanistic role of these ankyrin repeats remains less defined than that of the flanking BBD and PUFD domains. Although these repeats are highly conserved and may contribute to protein stability or multimerization, their specific binding partners and isoform-specific functions require further characterization.

As noted above, only a specific subset of BCOR alternative splice variants retains the internal AF9-binding domain, which enables these distinct isoforms to suppress AF9-mediated transcriptional activation in reporter assays [20]. Given that AF9 promotes transcriptional elongation and DOT1L-mediated H3K79 methylation, its sequestration by BCOR represents a proposed mechanism to restrain specific AF9-associated gene expression programs. In mouse progenitor and transplantation models of MLL-AF9 leukemia, disrupting the interaction between BCOR and MLL-AF9 altered the expression of developmental gene networks, including EYA and SIX family genes, underscoring a context-dependent role for BCOR in modulating AF9-driven transcription [44].

At its C-terminus, BCOR features the PUFD domain, which mediates direct binding to PCGF1 and ensures incorporation of BCOR into PRC1.1 [24]. X-ray crystallography defined the interface between the BCOR PUFD and PCGF1 RAWUL domains [24]. Solution nuclear magnetic resonance (NMR) analysis showed an ordered core and disordered N- and C-terminal regions in unbound BCOR PUFD [45]. In contrast, crystal structures demonstrate that these terminal regions form β-strands upon binding to PCGF1 [24]. These findings suggest a coupled folding-and-binding mechanism that forms an interface required for stable binding to the KDM2B-SKP1 heterodimer and subsequent PRC1.1 assembly [45].

### 3.3. Evolutionary Conservation of BCOR

Cross-species ortholog data from the Alliance of Genome Resources show that BCOR is highly conserved across vertebrates and invertebrates [46]. In *Drosophila melanogaster*, the BCOR-like protein CG14073 co-purified with Kdm2, SkpA, Sce, and l(3)73Ah within the fly PRC1.1-like Polycomb repressive complex [47]. This architectural preservation indicates that ancestral BCOR-related proteins were linked to the chromatin-modifying machinery long before the emergence of vertebrate-specific developmental adaptations. While the endogenous functions and precise developmental roles of CG14073 remain poorly characterized, its presence within a PRC1.1-like complex aligns with broader evolutionary models, suggesting that core PRC1 components originated early in eukaryotic evolution [48,49,50,51].

Studies in vertebrates have identified multiple developmental and transcription-factor-associated roles for BCOR. In humans, BCOR was originally identified by its interaction with the transcriptional repressor BCL6 [1]. Subsequently, deleterious germline *BCOR* mutations were shown to cause oculofaciocardiodental (OFCD) syndrome and related microphthalmia phenotypes [6]. Various animal models demonstrate the importance of BCOR in diverse developmental processes. In zebrafish, Bcor coordinates the morphogenesis of the eye, skeleton, and central nervous system [6]. In Xenopus, Bcor is required for establishing correct left–right patterning and embryonic cardiac orientation [7]. Furthermore, in mice, *Bcor* regulates early embryonic lineage programs and neural crest-derived craniofacial development [8,9,52]. Collectively, these comparative findings indicate that while BCOR preserves an evolutionarily ancient blueprint for epigenetic repression, it has adapted to drive lineage-specific developmental programs in vertebrates.

### 3.4. BCOR and BCORL1 in PRC1.1 Assembly and Chromatin Regulation

BCOR is a core component of PRC1.1, a distinct variant of the Polycomb repressive complex 1 (PRC1) [2,5]. This multiprotein machinery includes PCGF1, the E3 ubiquitin ligase catalytic core (RING1A or RING1B), the accessory protein RYBP or YAF2, the demethylase/DNA-binding protein KDM2B, the adaptor SKP1, and either BCOR or its paralog BCORL1 [2,3,4,5,36]. BCOR is incorporated into this complex through a highly specific interaction between its C-terminal PUFD domain and the RAWUL domain of PCGF1 [24]. X-ray crystallography and biochemical reconstitution showed that the BCORL1-PCGF1 heterodimer provides a binding interface for KDM2B [36]. These interactions generate a four-protein assembly module lacking the RING1 catalytic subunit [36].

Locus-specific genomic targeting of the holo-PRC1.1 complex is primarily driven by KDM2B, which directs the entire assembly to unmethylated CpG islands via its zinc finger CxxC (ZF-CxxC) DNA-binding domain [4,53]. Notably, while KDM2B contains a catalytic Jumonji C (JmjC) domain capable of mediating histone H3 lysine 36 (H3K36) demethylation, current mechanistic frameworks suggest that ZF-CxxC-dependent recruitment plays a far more important role in PRC1.1-mediated gene silencing than its enzymatic role as a demethylase [54,55]. Following chromatin recruitment, the activated RING1A/B-PCGF1 catalytic core deposits H2AK119ub1 [56,57]. This specific epigenetic modification can promote the recruitment of Polycomb repressive complex 2 (PRC2) to drive histone H3 lysine 27 trimethylation (H3K27me3), thereby generating a repressive feedback loop that supports Polycomb-mediated transcriptional repression [56,58].

Interestingly, functional studies in hESCs demonstrate that the recruitment of BCOR-PRC1.1 to a discrete subset of lineage-specific differentiation genes can occur independently of KDM2B [11]. Functional analysis of engineered BCOR mutants showed that while the BCOR C-terminus, which includes the linker and PUFD regions, mediates PRC1.1 assembly and chromatin recruitment, the N-terminus is required to preserve compact chromatin domains and actively repress BCOR target genes. The detailed assembly and downstream functions of PRC1.1 are schematically summarized in Figure 1B.

**Figure 1:**
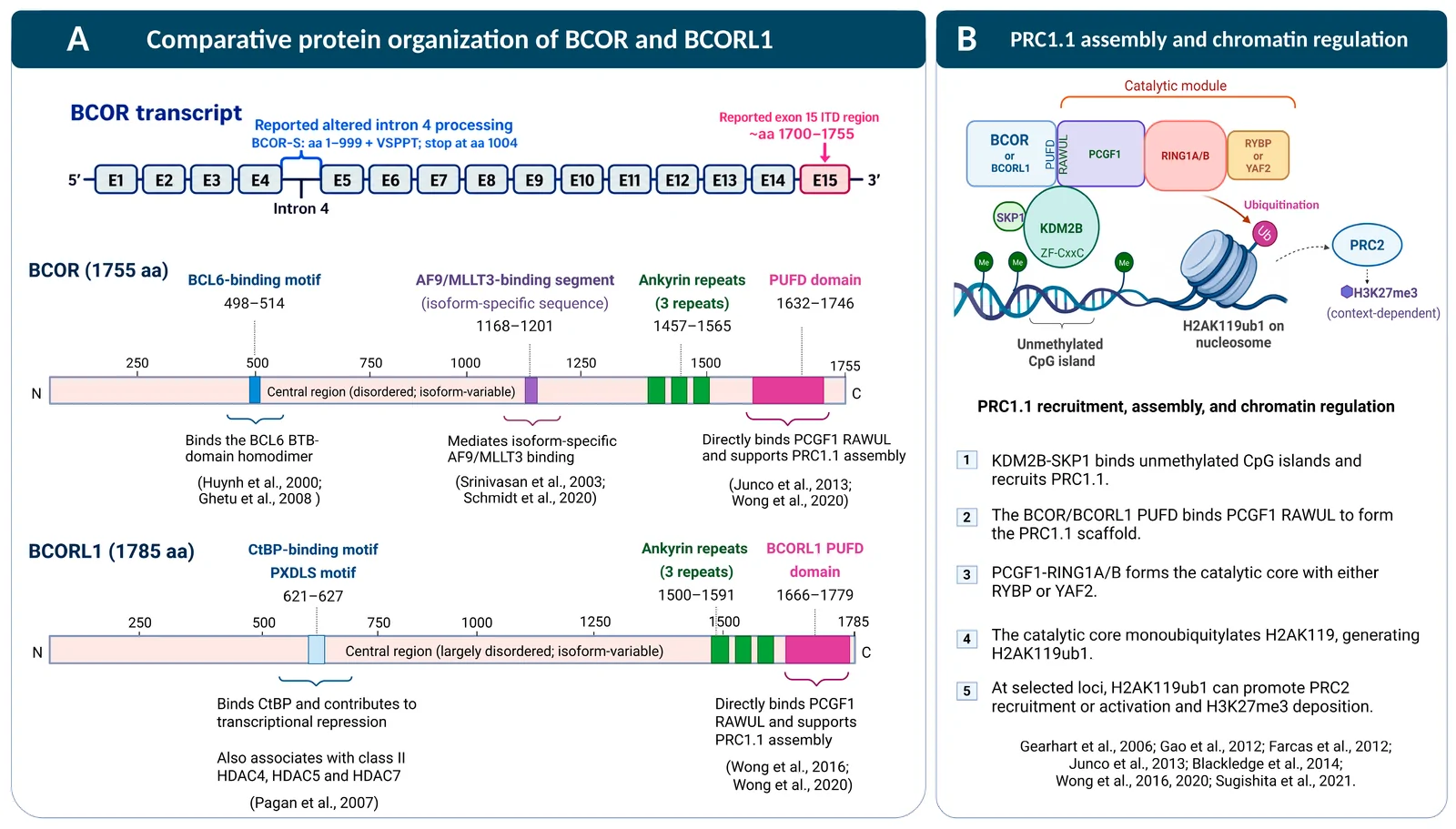
Gene and protein organization of BCOR and BCORL1 and assembly of PRC1.1. (**A**) *BCOR* exon schematic and comparison of the major functional regions of BCOR and BCORL1 proteins. The reference BCOR transcript is ENST00000378444.9 (BCOR-202), encoding 1755 amino acids. BCOR-S and exon ITD annotations summarize reported RNA-processing and duplication events [1,20,24,31,36,42,44,45]. Schematics are not to scale. (**B**) Simplified model of PRC1.1 recruitment, assembly, and chromatin regulation [2,4,5,24,36,56,58]. Dashed arrows indicate PRC2 recruitment and methylation. aa, amino acid. Created in BioRender.com.

## 4. BCOR in Cell Differentiation

### 4.1. BCOR in Pluripotency and Early Lineage Differentiation

Pluripotent stem cell models suggest that BCOR serves as a regulator of early lineage differentiation, though its explicit requirements differ between human and murine systems [9,11,52]. In hESCs, BCOR is a component of PRC1.1 required to maintain primed pluripotency [11]. Depletion of BCOR impaired ESC self-renewal, reduced PRC1.1 genomic occupancy at developmental loci, and triggered spontaneous differentiation toward endodermal and mesodermal lineages, marked by the emergence of FOXA2-positive and T/BRACHYURY-positive primitive streak precursors.

In contrast, a mouse ESCs (mESCs) study demonstrated that loss of full-length BCOR did not prevent self-renewal or pluripotency under baseline culture conditions [52]. Instead, phenotypic defects emerged primarily upon induction of multilineage differentiation via embryoid body assays. *Bcor*-deficient mESCs exhibited delayed transcriptional repression of the core pluripotency factor *Pou5f1* (Oct3/4), coupled with delayed induction of the early mesodermal and hematopoietic markers *T* (Brachyury) and *Flk1* [9]. In vivo chimeric mouse models generated from *Bcor*-deficient mESCs developed tail abnormalities, such as kinked or shortened tails, further suggesting the role of *Bcor* in the mesodermal lineage. Thus, while both human and murine models implicate BCOR as a necessary regulator of early lineage transitions, the precise temporal kinetics and directional lineage outcomes are species- and context-dependent.

### 4.2. BCOR in Hematopoietic Differentiation

BCOR is a critical determinant of early hematopoiesis, particularly for erythroid differentiation [9]. During mESC differentiation into embryoid bodies, loss of *Bcor* significantly delayed the transcriptional induction of pan-mesodermal and early hematopoietic markers (Brachyury and Flk1) and impaired the colony-forming capacity of primitive erythrocytes. In chimeric mouse models reconstituted with male *Bcor* loss-of-function mESCs, mutant cells exhibited a reduced contribution to B cells, T cells, and erythrocytes compared to wild-type controls, supporting a broader role for BCOR in hematopoietic development [9].

In definitive adult hematopoiesis, BCOR suppresses the aberrant expansion of myeloid-biased hematopoietic stem and progenitor cells (HSPCs) [59,60,61]. Conditional knockout and mutant *Bcor* models showed increased progenitor proliferation, lineage-biased myeloid differentiation, and the persistent reactivation of stem- and progenitor-associated transcriptional signatures. Mechanistically, *Bcor* ablation provoked locus-specific reductions in histone H2AK119ub1 deposition at key homeobox loci, including at *Hoxa7* and *Hoxa9*, leading to derepression of the *Hoxa* gene cluster and *Cebp* family of transcription factors. Studies using conditional Pcgf1 deletion in mice support a role for PRC1.1 in myeloid differentiation and myeloid progenitor self-renewal [62]. However, these findings do not establish that all effects of *Bcor* loss depend on PRC1.1. In primitive HSPCs, PRC1.1 acts to prevent the premature activation of C/EBPα-dependent myeloid differentiation programs, whereas in committed myeloid progenitors, it restrains HOXA9/β-catenin-driven self-renewal pathways to facilitate normal differentiation. Beyond the myeloid axis, BCOR maintains the balance between erythroid and megakaryocytic differentiation [63]. Inducible *Bcor* deletion in *Mx1-Cre* mice induced macrocytic anemia, increased platelet production, and expanded megakaryocyte-erythroid progenitors (MEPs) and megakaryocyte progenitors (MKPs). Collectively, these phenotypes demonstrate that BCOR regulates multiple stages of hematopoiesis, balancing primitive lineage commitment with control of adult myeloid and megakaryocytic progenitor pools.

### 4.3. BCOR in Lymphocyte Differentiation and Function

Within the adaptive immune system, the role of BCOR has been extensively characterized in the germinal center (GC) reaction during B cell development [1,64]. BCOR was first identified as a BCL6-interacting corepressor [1]. Subsequent studies showed that BCL6 and EZH2 cooperate to recruit and stabilize a CBX8-containing PRC1-BCOR complex at bivalent promoters in GC B cells [64]. This complex directly silences master regulators of plasma cell differentiation, such as *PRDM1* and *IRF4*, alongside key cell-cycle checkpoint genes including *CDKN1A* and *CDKN1B*. These findings establish a recruitment mechanism for this CBX8-containing PRC1-BCOR complex, but it is still unclear whether the same mechanism applies to all PRC1.1 complexes. By maintaining continuous epigenetic suppression at these loci, BCL6 and BCOR sustain GC B-cell proliferation and prevent premature termination during somatic hypermutation and affinity maturation.

BCOR also contributes to CD4^+^ T-cell differentiation [65]. T-cell-specific deletion of *Bcor* in mice reduced the number of GC T follicular helper (Tfh) cells following infection with *Listeria monocytogenes*. Similarly, in a peptide-immunization experiment, *Bcor* deficiency severely compromised T-cell-dependent B-cell differentiation, resulting in a parallel contraction of antigen-specific GC B cells and antibody-secreting plasma cells, indicating that intrinsic BCOR expression within T helper lineages is essential to sustain humoral immunity. Furthermore, loss of *Bcor* or *Kdm2b* in mouse T cells reduced T helper 17 (Th17) cell formation, supporting a possible involvement of PRC1.1 in this process [66]. Chromatin immunoprecipitation and expression analyses further supported BCOR-mediated repression of *Lef1*, *Runx2*, and *Dusp4*, which encode inhibitors of Th17 differentiation.

More recently, *Bcor* inactivation was shown to expand the proliferative pool of TCF1^+^ precursor-exhausted CD8^+^ T cells during chronic viral persistence [67]. Concomitant deletion of *Bcor* and *Zc3h12a*, which encodes the ribonuclease Regnase-1, synergistically enhanced CD8^+^ T cell function and improved viral control. These studies demonstrate that BCOR regulates the differentiation and function of diverse immune populations, including GC B cells, several CD4^+^ T-cell subsets, and CD8^+^ T cells [64,65,66,67].

### 4.4. BCOR in Neural Lineage Commitment and Neuronal Differentiation

BCOR coordinates several critical axes of neural differentiation, neurogenesis, and sensory organ development [68,69]. Within cerebellar granule neuron progenitors (CGNPs), BCOR forms a specialized transcriptional repressor complex alongside BCL6 and the NAD^+^-dependent deacetylase SIRT1 to restrict Sonic hedgehog (Shh) signaling [69]. These findings demonstrate BCOR-dependent repression. BCL6-BCOR-SIRT1 directly targets the regulatory regions of *Gli1* and *Gli2*, driving localized epigenetic silencing that downregulates their transcription and permits granule neuron differentiation to proceed. Furthermore, BCL6 also drives cortical neurogenesis by actively suppressing multiple pathways that sustain neural progenitor self-renewal, particularly Wnt and Notch signaling, as well as components of the Shh and Fgf pathways [68]. When BCL6 binds these targeted loci, it recruits SIRT1 to promote histone deacetylation and enforce transcriptional repression. However, whether BCOR or BCOR-containing PRC1.1 participates in this cortical repression program will require further investigation.

Studies using human pluripotent stem cells reinforce the baseline requirement for functional BCOR during early neuroectodermal commitment [70]. Large-scale genomic analysis identified a strong positive selection pressure for acquired truncating *BCOR* mutations during induced pluripotent stem cell (iPSC) culture, particularly in erythroblast-derived lines. Although these *BCOR*-mutant iPSCs preserved normal undifferentiated pluripotent morphology, they exhibited a severe defect upon neural differentiation. *BCOR* mutations resulted in fewer PAX6-positive neural stem/progenitor cells and reduced neuronal differentiation, alongside lower expression of *TUBB3*, *DCX*, and *FOXG1* and higher expression of mesoderm-associated genes. Whether these differentiation defects depend on PRC1.1 has not been established.

Recent mechanistic studies have linked BCOR-containing PRC1.1 to the deubiquitinase USP7, which is mutated in Hao-Fountain syndrome [71]. Studies using ESC-to-neural-stem-cell differentiation and SH-SY5Y neuroblastoma differentiation models showed that USP7 expression is essential for normal neuronal maturation. Genetic, biochemical, and chromatin analyses supported BCOR-PRC1.1 as a major downstream effector of USP7 in the SH-SY5Y model.

## 5. BCOR in Organ Development and Physiology

### 5.1. BCOR in Embryonic and Extraembryonic Development

BCOR is essential for normal mammalian development, with early evidence establishing an important role within extraembryonic tissues [9]. During mouse development, *Bcor* transcripts are highly expressed in extraembryonic tissues at E7.5–E8.5, particularly in the ectoplacental cone and trophectoderm, whereas expression within the embryo proper is relatively weaker. Following axial embryonic turning, *Bcor* expression broadens across the embryo proper, including the tail bud, branchial arches, limb buds, and neural tube [8].

Genetic studies in mice support this early developmental requirement [9]. In mice, the *Bcor^Neo^* loss-of-function allele showed a parent-of-origin effect. While male chimeras successfully transmitted the mutant allele through the germline, heterozygous females carrying the mutant allele failed to produce viable offspring that inherited the allele. Because the paternal X chromosome is selectively inactivated in mouse extraembryonic tissues, this inheritance pattern supports a requirement for functional BCOR in extraembryonic development. A subsequent X-linked gene-trap screen reinforced this early developmental role for BCOR [72]. Embryos generated entirely from two independent *Bcor* gene-trap mouse ESC lines failed to survive beyond E9.5, exhibiting severe developmental defects.

Recent conditional knockout mouse studies have successfully uncoupled these embryonic and extraembryonic requirements [52]. Conditional ablation of *Bcor* within epiblast-derived tissues using a *Sox2*-Cre driver did not recapitulate early embryonic lethality, provided a functional maternal allele remained intact within extraembryonic tissues. Conversely, targeted disruption of *Bcor* within extraembryonic lineages severely compromised placental architecture and parietal yolk sac development. Collectively, these findings establish a critical role for BCOR in the development of extraembryonic tissues and early post-implantation embryonic survival.

### 5.2. BCOR in Cardiac and Limb Development

BCOR is required for the morphogenesis of mesoderm-derived organs, including the heart and limbs [52]. Targeted conditional deletion of *Bcor* in mice within *Isl1*-expressing cardiovascular and limb progenitor cells caused mid-gestation fetal lethality. Mutant embryos displayed persistent truncus arteriosus (PTA), a severe heart defect characterized by the failure of the embryonic outflow tract to separate the major vessels leaving the heart into the aorta and pulmonary artery, as well as ventricular septal defects. Syndactyly involving the fusion of the second and third hindlimb digits was also observed. Furthermore, asymmetrical expression of *bcor* in *Xenopus tropicalis* is required for normal left–right axis specification [7]. Left-sided *bcor* depletion induced dextrocardia (reverse cardiac looping), abnormal gut rotational patterning, and reduced expression of the left-sided determinant *Pitx2c*. Mechanistically, the BCL6-BCOR complex repressed the Notch-responsive gene enhancer of split-related 1 (*ESR1*), which encodes a transcriptional repressor that would otherwise silence *Pitx2* expression within the left lateral plate mesoderm [73,74]. By actively repressing *Esr1* expression, BCL6 and BCOR helped maintain *Pitx2* expression during left–right patterning. These findings suggest a possible molecular explanation for the congenital laterality defects observed in a subset of patients with OFCD syndrome [7].

### 5.3. BCOR in Neural Tube and Craniofacial Development

In vivo animal models underscore the phenotypic consequences of *BCOR* knockdown and genetic deletion on neural and craniofacial development [6,52]. Knockdown of the *BCOR* ortholog in zebrafish provoked severe defects in neural tube closure, somite boundary formation, and eye development [6]. Corroborating these fish phenotypes, conditional knockout of *Bcor* within murine cranial neural crest cells caused cleft palate, mandibular hypoplasia, and glossal abnormalities, recapitulating the craniofacial features observed in human patients with OFCD syndrome [52].

### 5.4. BCOR in Retinal Function

BCOR serves as a critical regulator of visual function [12]. Within the mammalian retina, BCOR functions as a Polycomb-independent corepressor for the transcription factors CRX and OTX2 to modulate photoreceptor-specific gene expression networks. Furthermore, ectopic expression of selected patient-derived human BCOR mutants in mouse retinas, together with knockdown of endogenous *Bcor*, caused photoreceptor degeneration.

### 5.5. BCORL1 in Male Fertility and Its Possible Role in Neurodevelopment

Studies of BCORL1, a paralog of BCOR, have also examined its roles in development and physiology. In contrast to the absolute requirement of BCOR for early post-implantation embryonic survival, the reported *Bcorl1* frameshift-mutant mouse model did not exhibit early embryonic lethality [38]. In this model, generated by a CRISPR-mediated 22-bp frameshift deletion in exon 3 of *Bcorl1*, heterozygous females and hemizygous males were viable. Males were infertile, with impaired spermatogenesis, reduced sperm motility, and abnormal sperm mitochondrial structure. Complementary experiments in cultured mouse spermatogonial stem cells suggest that *Bcorl1* knockdown impairs self-renewal [38]. Additional human studies have reported hemizygous nonsense and missense variants in *BCORL1* in men with oligoasthenoteratozoospermia, further supporting a role for *BCORL1* in male fertility [75,76].

Beyond reproductive biology, hemizygous missense variants in *BCORL1* were reported in five individuals from three pedigrees with Shukla–Vernon syndrome, an X-linked neurodevelopmental disorder characterized by intellectual disability, dysmorphic features, and behavioral abnormalities [34]. A subsequent report described three patients from two unrelated families with hemizygous *BCORL1* missense variants, seizures, and major brain malformations, including polymicrogyria and abnormalities of the corpus callosum [39]. Both missense variants were classified as variants of uncertain significance, and their effects on BCORL1 expression and function were not experimentally established in that study [39]. A later diagnostic study also reported a hemizygous nonsense variant in an individual with neurodevelopmental impairment and epilepsy, indicating that reported human *BCORL1* variants are not restricted to missense alleles [40].

The missense variants described in the earlier reports should not be assumed to be functionally equivalent to the mouse *Bcorl1* frameshift allele [34,38,39]. Their effects could involve loss of function or altered activity, including gain-of-function, but these mechanisms remain unestablished. If a particular missense variant acts through gain-of-function, a knockout model may not reproduce its phenotype. Comparing knockout models with models carrying specific patient variants would therefore help distinguish these possibilities. Such comparisons would also require further characterization of the mouse neurological phenotype, as the original study reported abnormal brain development without presenting supporting data [38]. Taken together, current physiological and clinical evidence supports a role for BCORL1 in mouse male fertility and a possible role in human neurodevelopment. The developmental and physiological roles of BCOR and BCORL1 are summarized in Figure 2.

**Figure 2:**
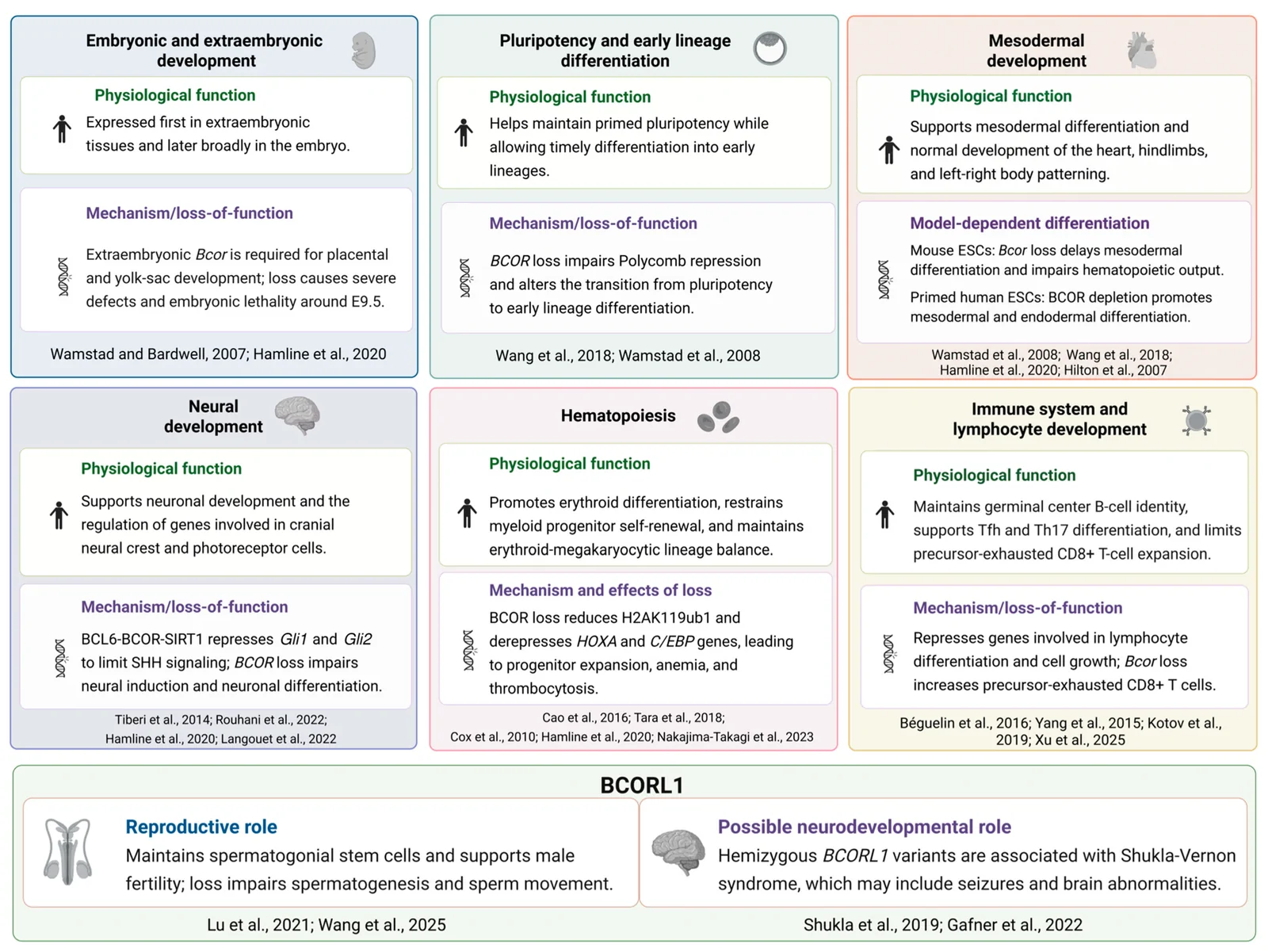
Developmental and physiological roles of BCOR and BCORL1. Early lineage differentiation outcomes depend on species, pluripotent state, and experimental conditions, as indicated in the relevant panel [7,8,9,11,12,34,38,39,52,59,61,62,64,65,66,67,69,70,72,76]. Abbreviations: H2AK119ub1, monoubiquitination of histone H2A at lysine 119; SHH, Sonic Hedgehog; Tfh, T follicular helper; Th17, T helper 17. Created in BioRender.com.

## 6. BCOR in Cancer

*BCOR* alterations are increasingly recognized across a wide spectrum of human malignancies; however, their downstream biological consequences depend on the type of alteration and the cellular lineage in which they arise. In hematologic malignancies and specific CNS neoplasms, *BCOR* generally functions as a tumor suppressor, and its inactivation by truncating mutations or other loss-of-function events can cooperate with other oncogenic alterations. In contrast, several pediatric and mesenchymal soft-tissue tumors are molecularly defined by recurrent genomic alterations involving *BCOR*. These include BCOR-ITDs and *BCOR* gene fusions, whose functional consequences remain incompletely defined. Studies of BCOR-ITD indicate context-dependent effects on Polycomb-associated regulation and differentiation, but its functional consequences in human tumors remain unresolved (Section 6.9). Cancers with *BCOR* alterations should therefore not be considered a single mechanistic group but interpreted according to the specific alteration, developmental and lineage context, and accompanying genetic changes. Table 2 summarizes the alteration types, proposed functional roles, co-alterations, and biological consequences across BCOR-associated tumors.

**Table 2:** Molecular features and evidence in BCOR-associated tumors. Abbreviations: BCOR-ITD, BCOR internal tandem duplication; CNS, central nervous system; ESC, embryonic stem cell; GC, germinal center; GNP, granule neuron progenitor; IGF1R, insulin-like growth factor 1 receptor; ITD, internal tandem duplication; NK/T, natural killer/T cell; PRC1.1, Polycomb repressive complex 1.1; SHH, Sonic Hedgehog.

Cancer Type	Alterations and Context	Key Findings and Limitations	References
Myeloid neoplasms	*BCOR*/*BCORL1* loss or truncation; selected *RUNX1*, *U2AF1*, *DNMT3A*, *or ASXL1* co-mutations	Cooperating tumor-suppressor loss. Loss/truncation can uncouple PCGF1-RING from KDM2B-bound targeting components, alter H2AK119ub1, and impair myeloid differentiation; supported by patient, chromatin, and mouse studies.	[25,37,59,60,61,63,77]
Lymphoid malignancies	Disruptive *BCOR* alleles; *MYC*- or *NOTCH1*-associated contexts	Mouse models support tumor suppression; BCOR-specific mechanisms in NK/T-cell lymphoma remain less defined	[78,79,80,81,82]
GC-derived B-cell lymphoma	Intact BCOR in models of BCL6/EZH2 activity	BCOR-associated repression supports GC maintenance; biochemical, genetic, and pharmacological evidence. Models were not selected for *BCOR* mutation	[64]
Sarcoma with *BCOR* genetic alterations (bone and soft tissue)	*BCOR::CCNB3*, BCOR-ITD, or rarer fusions; no consistent cooperating alteration established	Molecularly defining alterations; tumor cohorts and limited functional assays. PRC1.1 mechanism unresolved	[14,26,83,84,85,86,87]
High-grade endometrial stromal and related uterine sarcomas (*BCOR*/*BCORL1*-altered)	*BCOR*/*BCORL1* rearrangements or BCOR-ITD; *CDK4*/*MDM2* amplification or *CDKN2A/B* or *NF1* loss in subsets	Molecularly defining rearrangements/ITDs; direct oncogenic mechanism, tumor-initiation sufficiency, PRC1.1 effects, and drug dependencies remain unproven.	[41,88,89,90,91,92,93]
Clear-cell sarcoma of the kidney	BCOR-ITD in most cases; alternative *YWHAE::NUTM2* fusions	Diagnostic alteration with developmental/primitive nephrogenic signatures. Expression profiles suggest SHH/Hedgehog and AKT signaling, but functional dependence is unproven; *TP53* data are limited.	[15,94,95,96,97,98,99,100]
Retinoblastoma	Secondary *BCOR* loss in predominantly *RB1*-deficient disease	BCOR loss alters transcription; PRC1.1 dependence remains unresolved. Experimental models show context-dependent growth/stress-adaptation phenotypes and an IGF1R candidate dependency; clinical associations remain retrospective.	[101,102,103,104,105]
SHH medulloblastoma	*BCOR* loss/truncation, often C-terminally disruptive, in an SHH-prone background	Cooperates with *Ptch1* loss and derepresses *Igf2* in *Ptch1*-mutant mouse GNP models; direct chromatin/mouse evidence applies to the tested allele.	[106,107]
CNS tumor with BCOR-ITD	Somatic in-frame exon 15 BCOR-ITD; no consistent recurrent co-driver	Association with PRC1.1 components was retained in mouse ESC systems, with altered differentiation under EZH2 inhibition; tumor PRC1.1 dependence remains unresolved. SHH/WNT and IGF2-IGF1R-AKT are candidate pathways from limited models, including one patient-derived culture.	[16,17,108,109,110,111]

### 6.1. BCOR and BCORL1 in Myeloid Malignancies

Recurrent somatic mutations in *BCOR* and *BCORL1* have been reported in acute myeloid leukemia (AML), myelodysplastic syndromes (MDS), and other related myeloid neoplasms [13,25,35]. Within the AML and MDS cohorts, *BCOR* mutations are predominantly loss-of-function alterations, including frameshift, nonsense, and splice-site mutations that reduce or abolish BCOR protein function [13,25,112]. These mutations frequently co-occur with other myeloid-associated mutations, including those in the transcription factor *RUNX1*, the splicing factor *U2AF1*, and the epigenetic regulators *DNMT3A* and *ASXL1* [25,77,113]. *BCOR* mutations may be acquired at later steps of the oncogenic cascade, suggesting that they are not the tumor-initiating mutation but do contribute to tumor progression once a tumor has started [25]. This interpretation is consistent with broader evidence for clonal evolution in AML and MDS [114,115,116,117,118].

Mechanistic investigations demonstrate that *BCOR* and *BCORL1* mutations can impair PRC1.1-mediated transcriptional repression, with direct complex-level evidence in human myeloid models [37,59,60,61]. In mouse models, reduced BCOR function increases the progenitor population, alters myeloid differentiation, and derepresses BCOR-regulated lineage and self-renewal genes, including Hoxa-family genes such as *Hoxa7* and *Hoxa9* [59,60,61]. These changes in differentiation and transcription appear to promote disease progression when additional mutations are present. *Bcor* depletion produced more severe MDS-like disease when *Tet2* was lost [61]. In another context, *Bcor* loss promoted AML-like disease when an oncogenic *Kras* was present [60]. In human myeloid models, protein-interaction assays, ChIP-seq analyses, and rescue experiments showed that BCOR/BCORL1 loss or BCOR C-terminal truncation can uncouple the PCGF1-RING catalytic module from KDM2B-containing targeting components, impairing repression and activating oncogenic signaling. Targeting components can remain chromatin-bound, and BCORL1-containing complexes can retain activity after BCOR loss [37].

Collectively, these molecular networks outline a tumor-suppressor role for BCOR and BCORL1 as components of PRC1.1 in myeloid neoplasms [37,59,60,61,63]. *BCOR* or *BCORL1* disruption derepresses developmental, self-renewal, and signaling pathways and alters progenitor differentiation and proliferation in models of MDS, AML, erythroid leukemia, or myelofibrosis. However, PRC1.1-independent mechanisms have not been ruled out and warrant further investigation. Clinically, *BCOR* mutations are associated with adverse-risk biology in AML [112]. While no clinically approved BCOR-directed therapeutic agents currently exist, preclinical investigations suggest that BCOR/BCORL1-PRC1.1 disruption may alter therapeutic sensitivity [37]. In K562 models, PRC1.1 disruption promoted FGFR1-mediated imatinib resistance, which was reversed by combined imatinib and fibroblast growth factor receptor (FGFR) inhibition [37]. Loss of PRC1.1 components, including BCOR, also conferred menin-inhibitor resistance to preclinical *KMT2A*-rearranged AML models [119,120]. Whether PRC1.1 loss predicts resistance in patients remains unclear.

### 6.2. BCOR in Lymphoid Malignancies

Somatic *BCOR* aberrations are frequently identified across various lymphoid malignancies, though their functional contributions are context-dependent and lineage-specific compared to their tumor-suppressor role in myeloid oncology [79,82,121,122]. In mature human B-cell lymphomas, recurrent *BCOR* loss-of-function mutations are candidate drivers identified through genomic profiling in splenic diffuse red pulp small B-cell lymphoma [122]. Genomic profiling has identified recurrent *BCOR* mutations in diffuse large B-cell lymphoma (DLBCL), especially in the activated B-cell-like (ABC) subtype [121]. Functional validation in genetically engineered mouse models confirms that BCOR acts as a classic tumor suppressor in several models of mature B-cell malignancies [79,82]. Within the Eμ-*Myc* transgenic mouse model, a classic B-cell lymphoma model driven by constitutive *Myc* overexpression, the loss of *Bcor* significantly accelerated high-grade lymphoma onset, establishing its potent in vivo tumor-suppressor capacity. Similarly, in mature B-cell neoplasms, *Bcor* inactivation drove the malignant transformation of indolent chronic lymphocytic leukemia (CLL) into an aggressive, lethal Richter-like transformation phenotype [82]. This histopathological progression was characterized by increased Notch1 signaling and changes in the tumor microenvironment, specifically an increase in PD-1-positive T cells [82]. However, because this phenotypic transformation relies on the background context of the Eμ-*Tcl1* CLL mouse model, *Bcor* loss functions as an essential cooperating alteration during CLL progression rather than an independent initiator of mature B-cell malignancies.

Experimental studies support a tumor-suppressor role for BCOR in mouse T-cell malignancies, while evidence in NK/T-cell malignancies is based largely on mutation analyses of patient tumors [78,80]. Conditional deletion of *Bcor* exon 4 in mouse hematopoietic cells produced a BCOR protein lacking the BCL6-binding region, increased thymocyte proliferation in culture, and predisposed mice to T-cell acute lymphoblastic leukemia (T-ALL), largely through Notch1-dependent mechanisms. Chromatin analyses identified BCOR occupancy at the *Myc* promoter and other Notch1 target promoters in mouse thymocytes. The BCOR ΔE4 protein retained association with PCGF1 and RING1B; so, this phenotype should not be attributed to PRC1.1 disruption alone [80]. Separately, *Kdm2b*-deficient mouse models support a role for PRC1.1 in restraining Notch1-driven T-cell leukemogenesis [123]. Recurrent loss-of-function *BCOR* mutations have been reported in extranodal NK/T-cell lymphoma [78]. These highly aggressive, Epstein-Barr virus (EBV)-associated malignancies typically feature a complex mutational landscape, with co-occurring mutations within the JAK/STAT signaling pathway, the RNA helicase *DDX3X*, the tumor suppressor *TP53*, the transcription factor *MGA*, the histone methyltransferase *KMT2D*, and other epigenetic or immune-regulatory genes [78,124,125]. MYC overexpression is present in extranodal NK/T-cell lymphoma, and *BCOR* mutations have been associated with high MYC expression and poor prognosis in an observational tumor cohort [81,126,127].

Conversely, germinal center (GC) B cell-derived lymphomas provide an important exception to the tumor-suppressor role of BCOR [64]. In this context, unmutated BCOR supports lymphomagenesis as part of a BCL6/EZH2-dependent PRC1-BCOR-CBX8 complex, a specialized assembly whose findings should not be generalized to all PRC1.1 complexes. This complex is recruited to bivalent promoters and represses genes involved in plasma-cell differentiation (*PRDM1* and *IRF4*) and the cell cycle (*CDKN1A* and *CDKN1B*). By maintaining continuous epigenetic repression at these loci, the functional complex sustains GC B-cell proliferative capacity and stalls terminal differentiation during affinity maturation. Corroborating this story, conditional deletion of *Bcor* or genetic disruption of the BCL6-BCOR interaction blocked GC hyperplasia and slowed lymphoma progression. For the treatment of diffuse large B-cell lymphoma models, dual pharmacological inhibition targeting both EZH2 and BCL6 showed greater anti-lymphoma activity than either agent alone in cell cultures, cell-line xenografts, and four primary germinal center B-cell-type (GCB) DLBCL samples tested ex vivo.

Collectively, these studies underscore that BCOR has context-dependent functions in lymphoid oncology [64,79,80,82]. It acts as a barrier to transformation in MYC- or NOTCH1-driven settings, while serving as a survival factor when embedded within BCL6/EZH2-driven germinal center malignancies.

### 6.3. Sarcomas with BCOR Genetic Alterations in Bone and Soft Tissue

Sarcomas with *BCOR* genetic alterations constitute a molecularly defined category of bone and soft-tissue neoplasms that are histopathologically and genetically distinct from classic Ewing sarcoma, *CIC*-rearranged sarcomas, and other round-cell sarcoma entities [14,26,83,85,87]. The defining recurrent alterations in this family include the *BCOR::CCNB3* fusion, BCOR-ITD, and rarer *BCOR* chimeric configurations such as *BCOR::MAML3* and *ZC3H7B::BCOR*. Unlike myeloid neoplasms, recurrent cooperating mutations are less well defined in these sarcomas [83,85,128]. Instead, current research focuses on characterizing *BCOR* gene fusions and ITDs and their associated transcriptional or epigenetic profiles.

The *BCOR::CCNB3* sarcoma represents a highly characterized subtype originally identified as an “Ewing-like” sarcoma lacking canonical *EWSR1-ETS* gene rearrangements [14]. The resulting fusion oncoprotein retains nearly the entire BCOR amino acid sequence and incorporates a CCNB3-derived C-terminal segment [14,129]. Mechanistically, *BCOR::CCNB3* sarcomas show altered gene-expression and DNA-methylation profiles [83,85,94]. This subtype shares transcriptional features with other malignancies carrying *BCOR* alterations, including upregulation of *BCOR* transcripts, PRC2-regulated gene networks, and specific homeobox (HOX) clusters. Multi-omics profiling of one *BCOR::CCNB3* renal sarcoma identified altered expression of chromatin regulators and DNA methylation patterns [128]. In NIH3T3 cells, overexpression of *BCOR::CCNB3* or truncated *CCNB3* increased the proportion of cells in S phase [14]. This finding does not establish cell-cycle checkpoint activation or the mechanism of sarcoma initiation. Whether *BCOR::CCNB3* fusion alone is sufficient to initiate mesenchymal transformation in human tissues remains unclear.

BCOR-ITD sarcomas represent another major clinical entity within this sarcoma family [26,84]. Distinct pediatric malignancies, including primitive myxoid mesenchymal tumors of infancy (PMMTI) and undifferentiated round-cell sarcomas (URCS) of infancy, are now unified within this broader BCOR-ITD soft-tissue tumor spectrum. These neoplasms have a localized duplication within the highly conserved C-terminal PUFD domain of BCOR, correlating with increased expression of *BCOR* transcripts and protein, which has prompted neomorphic or hypermorphic hypotheses but does not establish either mechanism [26,84]. These tumors share morphological, immunohistochemical, and molecular features with clear-cell sarcoma of the kidney (CCSK) [26,86]. However, most BCOR-ITD sarcoma research to date focuses on establishing a diagnostic molecular classification and expression patterns. Direct mechanistic studies using dedicated BCOR-ITD model systems are still urgently needed to determine precisely how BCOR-ITD modifies Polycomb-dependent epigenetic regulation.

In summary, sarcomas with *BCOR* alterations appear to represent a distinct tumor family in which *BCOR* gene fusions and ITDs are associated with developmentally primitive transcriptional profiles [83,85,94,128]. Nevertheless, the depth of mechanistic evidence varies from mutation to mutation. *BCOR::CCNB3* sarcomas have been studied using transcriptomic profiling and, in one case, multi-omics analysis showing altered gene expression and DNA methylation. In contrast, soft-tissue BCOR-ITD sarcomas have been characterized mainly through molecular classification and diagnostic biomarker profiles. Therapies targeting BCOR mutants in these sarcomas are currently not available. At present, the primary clinical utility of *BCOR* alterations remains centered on diagnostic classification, while ongoing functional studies aim to define the downstream pathways that drive tumor growth as potential therapeutic targets [17,29,85].

### 6.4. BCOR and BCORL1 Alterations in Endometrial Stromal and Related Uterine Sarcomas

Genetic alterations in *BCOR* are characteristic features found in high-grade endometrial stromal sarcoma (HG-ESS) and related uterine sarcomas, including the *ZC3H7B::BCOR* fusion, BCOR-ITD, and variant *BCOR* or *BCORL1* rearrangements [41,88,93,130,131]. The best-characterized alteration is *ZC3H7B::BCOR*, which results from a chromosomal translocation between chromosome 22 and the X chromosome and was first identified in endometrial stromal sarcoma [130]. BCOR-ITD has also been reported in HG-ESS [92,132]. These alterations are diagnostically important because uterine sarcomas with *BCOR* alterations can morphologically resemble myxoid leiomyosarcoma, undifferentiated uterine sarcoma, or other high-grade uterine mesenchymal tumors [88,131,133].

How *BCOR* alterations promote tumor formation in these uterine sarcomas remains undefined. *ZC3H7B::BCOR* is unlikely to act as a simple *BCOR* loss-of-function mutation because the predicted fusion protein retains several regulatory regions, including the AF9-binding region, ankyrin repeats, and the C-terminal PUFD domain required for PCGF1 interaction [130]. Conversely, the BCOR-ITD mutation is a distinct alteration that directly affects the PUFD region, which is essential for PRC1.1 assembly [24]. The involvement of BCOR interaction regions raises the possibility that these fusions and ITDs alter PRC1.1-associated transcriptional repression, although this has not been directly tested in uterine sarcoma models. Epigenetic and transcriptional profiling, which does not directly establish PRC1.1 involvement, shows that HG-ESS with *BCOR* alterations is molecularly distinct from low-grade endometrial stromal sarcoma and clusters within a broader group of high-grade uterine sarcomas [93,134].

Recurrent co-alterations may help explain how these tumors grow and progress [89,90,93]. In *BCOR*-rearranged uterine sarcomas, recurrent copy-number changes include *CDK4* amplification, loss of *CDKN2A/B*, *MDM2* amplification, and, in a subset of cases, loss of *NF1* [89,93]. Similar co-alterations have also been reported in uterine sarcomas with *BCORL1* alterations, including *CDK4* amplification, *CDKN2A* loss, *NF1* mutations, and changes affecting the NF2-mTOR pathway [90]. These recurrent alterations disrupt cell-cycle regulation, the p53 pathway, and RAS/MAPK or mTOR signaling, which may drive tumor growth in selected cases. Currently, most studies focus on diagnostic, clinicopathologic, methylation-oriented, or genomic profiling. No relevant functional model has yet demonstrated that *ZC3H7B::BCOR* alone is sufficient to initiate tumorigenesis. Consequently, no BCOR-directed therapy has been established for these tumors, but recurrent alterations affecting *CDK4*, *MDM2*, *NF1*, and related pathways may provide targeted therapeutic opportunities in selected cases.

### 6.5. BCOR Alterations in Clear-Cell Sarcoma of the Kidney and Related Renal Tumors

Clear-cell sarcoma of the kidney (CCSK) is one of the most well-established BCOR-ITD tumors [15,94,135]. Recurrent in-frame ITDs within the PUFD domain of BCOR are identified in the vast majority of CCSK cases. These BCOR-ITD mutations are associated with a high expression of both mutant *BCOR* transcripts and protein products, although expression alone does not establish the functional effect of BCOR-ITD [94,135]. Consequently, BCOR-ITD serves as a robust diagnostic molecular marker to differentiate CCSK from Wilms tumor and other pediatric renal malignancies, particularly for those with overlapping histomorphology [15,135]. CCSK generally has a relatively simple genomic profile, and consistent recurrent cooperating alterations remain incompletely defined [98,135].

While most CCSK cases harbor the characteristic BCOR-ITD, rare non-ITD cases with *YWHAE::NUTM2B/E* or *BCOR::CCNB3* gene fusions have also been documented [95,99,136]. Although these tumors are genetically distinct from the predominant BCOR-ITD subgroup, the degree to which they represent biologically or clinically unique CCSK entities remains less established. Epigenetically, CCSK exhibits a distinct DNA methylation profile that distinguishes it from other pediatric renal tumors [137]. Subsequent investigations have demonstrated global CpG-island hypermethylation and EZH2 overexpression across primary CCSK molecular subgroups, including both BCOR-ITD and *YWHAE::NUTM2B/E*-positive cases [95]. Furthermore, CCSK shows a marked reduction in the expression of TCF21, a transcription factor involved in mesenchymal and renal development [98]. This downregulation is linked to hypermethylation of the *TCF21* promoter alongside low expression of *TARID*, a regulatory long noncoding RNA (lncRNA) that maintains TCF21 expression.

Mechanistically, BCOR-ITD is hypothesized to modify PRC1.1-mediated transcriptional repression [94]. BCOR-ITD-positive CCSK displays a significant upregulation of PRC2-target, homeobox, and developmental gene programs, reflecting transcriptional commonalities with other sarcomas carrying *BCOR* alterations. Transcriptomic profiling has revealed the upregulation of neural-developmental genes such as *NPTX2*, *NTRK3*, and *NGFR*, alongside the induction of *GLI1* and *PTCH1*, which suggests increased Sonic hedgehog-associated transcription without establishing pathway dependence [96]. Similarly, WNT-related developmental genes (*WNT5A* and *WNT11*) were dysregulated, and PI3K-AKT pathway components (*FOXO1* and *PDGFRA*) exhibited altered expression patterns. Furthermore, CCSK retains primitive embryonic renal markers, including *OSR1*, *FOXD1*, and *CITED1* [97]. Taken together, these findings suggest that BCOR-ITD may alter Polycomb-mediated repression and contribute to the primitive nephrogenic or intermediate-mesoderm-like expression state reported in CCSK [94,97]. However, these studies do not establish PRC1.1 dependence or a BCOR-ITD-induced differentiation block. Although a BCOR-ITD variant impairs PCGF1 binding and PRC1.1 module assembly in biochemical assays [45], how this alteration affects PRC1.1 in CCSK cells remains to be determined.

### 6.6. BCOR Loss in Retinoblastoma

Retinoblastoma is primarily an *RB1*-driven malignancy, with most cases arising following the biallelic functional loss of *RB1* within retinal precursor cells of the cone lineage [102,103,138]. In a subset of these tumors, *BCOR* alterations occur as recurrent secondary loss-of-function events, presenting predominantly as truncating or otherwise damaging mutations [101,102,103,138]. Additional genomic alterations involving cell-cycle regulators and growth-signaling genes have been reported across retinoblastoma cohorts [101,102,103,138]. Their frequency and functional cooperation specifically in BCOR-mutant tumors remain incompletely defined. Furthermore, specific cohorts of patients exhibit characteristic changes in somatic copy-number alterations, including chromosomal 1q, 6p, and 17q gains and loss of 16q.

Functional studies suggest that BCOR deficiency drives retinoblastoma progression via at least two pathways: enhanced hypoxic adaptation following BCOR loss, for which PRC1.1 dependence remains unresolved, and upregulation of insulin-like growth factor 1 (IGF1) signaling, which supports tumor growth, invasion, and chemoresistance [104,105]. Within retinoblastoma cells, BCOR associated with core components of the PRC1.1 complex; loss of BCOR downregulated cell-cycle gene expression while upregulating genes involved in adaptation to hypoxia [105], suggesting that BCOR-deficient cells transition into a stress-tolerant state. This hypoxia-tolerant state may also be linked to the RB1-ESRRG-mediated cell survival pathways under low-oxygen conditions [105,139]. While ESRRG promoted retinoblastoma cell survival during hypoxia, BCOR could actively repress ESRRG-driven transcription independently of RB1. In parallel, experimental BCOR depletion in retinoblastoma cells increased IGF1 expression and activates IGF1R [104]. These cells showed increased sensitivity to linsitinib and AEW541 in vitro. Linsitinib also inhibited tumor growth in xenograft models. However, clinical benefit in patients with BCOR-deficient retinoblastoma remains unvalidated. Clinically, *BCOR*-mutant retinoblastomas exhibit aggressive histopathologic features and diminished metastasis-free survival [103,104], though current clinical data have not established BCOR loss as an independent cause of distant metastasis.

### 6.7. BCOR Loss in SHH-Activated Medulloblastoma

In sonic hedgehog (SHH)-activated medulloblastoma, recurrent somatic *BCOR* alterations are predominantly loss-of-function mutations that occur more frequently in the SHH subgroup compared to other molecular subgroups of medulloblastoma [106,107]. In the mouse model discussed below, *Ptch1* loss provides the cooperating SHH-pathway alteration [107]. In mechanistic murine models, the targeted deletion of *Bcor* exons 9–10 alone was insufficient to induce tumorigenesis or perturb the baseline differentiation of granule-neuron progenitors. However, combining *Bcor* ΔE9–10 with heterozygous loss of *Ptch1* generated fully penetrant medulloblastomas with a significantly shorter time to tumor development in this mouse model. This genetic context is critical because SHH medulloblastomas originate from granule-neuron progenitors, which are particularly responsive to the SHH pathway [140]. Therefore, *Bcor* loss-of-function is best conceptualized as a cooperating oncogenic event in SHH-prone cerebellar precursors rather than a primary tumor-initiating driver.

In the *Ptch1*-mutant mouse model, C-terminal *Bcor* truncation reduced BCOR occupancy and H2AK119ub1 at the insulin-like growth factor 2 (*Igf2)* promoter and was associated with *Igf2* derepression [107]. Ectopic expression of IGF2 was sufficient to transform *Ptch1*^+/−^ preneoplastic granule-neuron progenitors. This finding was supported by earlier SHH-activated medulloblastoma studies demonstrating that IGF2 promoted *Ptch1*-mutant tumorigenesis by regulating the proliferation of cerebellar precursor cells, serving as a critical checkpoint for the progression of preneoplastic lesions into advanced medulloblastoma [141,142,143]. Furthermore, as described in preceding sections, the BCL6/BCOR/SIRT1 complex can directly silence downstream SHH effectors, including *Gli1* and *Gli2*, thereby suppressing medulloblastoma formation [69]. Taken together, these data suggest that BCOR suppresses IGF2 through PRC1.1-associated chromatin repression and SHH effectors through the separately studied BCL6-BCOR-SIRT1 complex. Whether baseline *BCOR* mutation status modifies clinical responses to existing SHH-directed therapies remains to be established.

### 6.8. CNS Tumor with BCOR-ITD

#### 6.8.1. Molecular Classification and Pathological Features

CNS tumor with BCOR-ITD is a rare pediatric embryonal malignancy defined by a recurrent in-frame ITD mutation in exon 15 of the *BCOR* gene [16,28]. This entity was identified as a subgroup of the central nervous system primitive neuroectodermal tumor (CNS-PNET) category through genome-wide DNA methylation profiling, which identified a distinct, molecularly defined entity designated as CNS HGNET-BCOR [16]. The 2021 WHO classification later recognized this entity as “CNS tumor with BCOR internal tandem duplication” within the broader category of CNS embryonal tumors [28]. This reclassification is of vital diagnostic importance; diagnosis is no longer dictated by histomorphology alone and requires molecular confirmation of the defining *BCOR* exon 15 ITD. Genome-wide DNA methylation profiling provides robust diagnostic support by confirming the characteristic methylation class [16,28].

From an immunohistochemical perspective, CNS BCOR-ITD tumors typically display diffuse, robust nuclear BCOR immunoreactivity, establishing BCOR immunohistochemistry (IHC) as an effective initial screening tool [17,144]. However, standalone BCOR expression cannot definitively establish a diagnosis or confirm the underlying genomic alteration [145,146]. Strong nuclear BCOR positivity also occurs in other CNS tumors, such as tumors harboring the *EP300::BCOR* gene fusion or tumors reported as CNS HGNET-BCOR without exon 15 ITD [146]. In addition to BCOR, these tumors frequently express SATB2, cyclin D1, BCL2, and TLE1, whereas immunoreactivity to vimentin, CD56, and CD99 has been reported in smaller series but remains highly nonspecific [17,144]. Expression of glial markers like GFAP and OLIG2 is variable, frequently presenting as entirely absent or limited to focal subpopulations [17,144,147]. Nonetheless, a cohort of patients exhibits diffuse GFAP and S100 protein expression accompanied by clear morphological evidence of glial differentiation. The expression profile of neuronal-lineage markers is similarly heterogeneous [17,144,148,149,150]. Across documented cohorts, NeuN and neurofilaments are more frequently detected than synaptophysin, though staining patterns remain focal or confined to minor tumor subpopulations, with several molecularly confirmed cases remaining immunonegative for classical neuronal markers.

Recurrent somatic co-mutations or secondary driver alterations have not been definitively established in CNS tumors with BCOR-ITD [16,149]. The majority of reported cases exhibit relatively simple copy-number and genomic profiles. However, homozygous deletion of the tumor suppressor gene *CDKN2A* was identified in two cases within the original cohort [16]. Subsequent sequencing efforts have revealed sporadic, isolated alterations involving genes such as *TP53*, *BCORL1*, *STAG2*, *TERT*, and *SMARCA2*; however, these aberrations remain restricted to individual cases and have not been replicated as recurrent oncogenic co-drivers [149]. A germline *PTCH1* single nucleotide polymorphism (SNP) (rs357564) was reported in one patient, though its direct functional contribution to the SHH pathway activation remains uncertain [108]. Collectively, these findings identify BCOR-ITD as the defining recurrent alteration in these tumors, while reported secondary alterations are uncommon and heterogeneous, without an established consistent co-driver.

#### 6.8.2. Effects of BCOR-ITD on Polycomb Regulation and Differentiation

The effects of BCOR-ITD differ across experimental models, and how it contributes to tumor development remains unclear [110,111]. In human neural stem cell models, the ectopic expression of full-length BCOR-ITD resulted in the derepression of Polycomb repressive complex 2 (PRC2)/H3K27me3-linked developmental targets, including *PITX1*, *WNT11*, and *GATA6* [110]. However, full-length BCOR-ITD expression did not confer a stable, long-term growth advantage to human neural stem cells, and sustained transgene expression could not be maintained over extended passages. A truncated *BCOR* E7–15 ITD construct exhibited a more pronounced oncogenic phenotype in murine neural stem cells, including increased cell proliferation, invasion, and migration alongside significantly reduced global levels of H2AK119ub1, a change that does not by itself establish PRC1.1 involvement because several PRC1 complexes generate this mark. These data suggest context-dependent effects of BCOR-ITD on Polycomb-linked regulation and growth in vitro, albeit tumor initiation in vivo was not tested.

Studies in mouse embryonic stem cells using transgenic BCOR-ITD expression and endogenous *Bcor*-ITD knock-in systems showed retained PCGF1 binding and association with PRC1.1 components [111]. These cells showed no distinct growth advantage under standard culture conditions. During directed differentiation with EZH2 inhibition, however, they developed an abnormal CD24/CD26 double-positive subpopulation, indicating an altered differentiation response in this sensitized setting. The possible mechanisms and limitations of these findings are discussed in Section 6.9.

#### 6.8.3. Candidate Signaling Pathways in CNS Tumors with BCOR-ITD

SHH/GLI and canonical WNT/β-catenin signaling have been implicated in CNS tumors with BCOR-ITD through tumor profiling and limited experimental studies [16,108]. Transcriptomic pathway analysis of primary and matched metastatic specimens from one patient revealed a basal cell carcinoma-like signature associated with SHH/GLI and canonical WNT signaling [108]. Upregulation of *GLI1* and *SMO* transcripts supports elevated GLI-associated transcription, while nuclear β-catenin and elevated *AXIN2* and *LEF1* expression support canonical WNT signaling. Direct activation of either pathway by BCOR-ITD remains unproven. These specimens also exhibited strong induction of *IGF2* coupled with modest upregulation of *IGF1R*.

High *IGF2* and *IGF1R* transcript levels were reported in tumor samples from three patients and in the metastatic patient-derived PhKh1 culture [109]. Functional studies in PhKh1 supported IGF1R-AKT signaling. Because the same patient-derived culture was used in the earlier SHH/WNT studies [108], additional independent models are needed to determine how consistently this pathway contributes to CNS tumors with BCOR-ITD. Pharmacological findings are discussed in Section 7.2.

A subset of CNS BCOR-ITD tumors displays increased expression of the receptor tyrosine kinases (RTKs) *PDGFRA* and *NTRK2* alongside the neurotrophin co-receptor *NGFR* and the WNT ligand *WNT7B* [17]. Robust EGFR protein expression was observed in CNS BCOR-ITD cohorts. A conference abstract examined four CNS cell models from three patients with different BCOR alterations. The two BCOR-ITD models came from one patient [151]. Ligand stimulation with basic fibroblast growth factor (bFGF) or PDGF-AA increased ERK, AKT, and S6 phosphorylation, consistent with FGF/PDGF-responsive signaling in these models. Collectively, these findings support FGF/PDGF signaling as a candidate contributor to growth; however, receptor-specific validation across a larger panel of tumor models is required to determine whether these receptors are therapeutic targets across independent models. The major candidate signaling pathways reported in CNS tumors with BCOR-ITD, along with their current levels of experimental and clinical validation, are summarized in Figure 3.

Overall, accumulated evidence links BCOR-ITD to altered Polycomb-associated gene expression and context-dependent differentiation phenotypes, with retained association with PRC1.1 components in mouse ESC systems. Tumor profiling and limited functional studies also implicate growth-related signaling, but the causal links to PRC1.1 remain unresolved [17,108,109,110,111,151]. Despite these mechanistic insights, the field still lacks a broad panel of patient-derived cell lines, stable full-length BCOR-ITD tumor models, robust in vivo animal models, and reproducible data supporting targeted therapies. Consequently, whether BCOR-ITD acts as a standalone driver sufficient to fully recapitulate the malignant tumor phenotype remains to be definitively established. Pharmacological findings involving arsenic trioxide (ATO), IGF1R inhibitors, and multikinase inhibitors, together with their experimental and clinical limitations, are discussed in Section 7.

### 6.9. Mechanistic Interpretation Across BCOR Alteration Classes

*BCOR* genomic alterations may affect protein function in different ways. *BCOR* loss or PUFD-disrupting truncation impairs PRC1.1-mediated repression by disrupting the association between its catalytic and targeting components, while BCORL1-containing PRC1.1 complexes remain intact and provide residual PRC1.1 activity [37]. In mouse myeloid models, reduced BCOR function expands progenitor populations, alters differentiation, and derepresses lineage-specifying and self-renewal genes [59,60,61]. In selected models, reduced BCOR function or C-terminally disruptive *Bcor* alleles cooperate with additional oncogenic alterations, including *Tet2* loss, oncogenic *Kras*, and *Ptch1* loss [60,61,107]. Although high BCOR expression has been reported in several *BCOR*-altered tumors [26,85,94,110], and enrichment of PRC2-associated transcriptional programs has been reported in BCOR-ITD tumors and experimental models [94,110], these observations alone do not distinguish among loss-of-function, hypermorphic, neomorphic, or dominant-negative mechanisms.

For BCOR-ITD, experimental findings differ between recombinant protein-fragment assays and cellular models. In recombinant protein-fragment experiments, a 30-amino-acid duplication in the PUFD domain impaired PCGF1 binding and complex assembly [45]. In contrast, full-length BCOR-ITD in mouse ESCs did not disrupt interaction with PCGF1 but showed abnormal differentiation under EZH2 inhibition [111]. Full-length BCOR-ITD increased expression of developmental and PRC2-associated target genes in human neural stem cells, whereas a truncated BCOR E7–15 ITD construct was associated with reduced global H2AK119ub1 in mouse neural stem cells [110]. Differences in protein constructs and assay conditions may contribute to these findings, but the basis for the discrepancy remains unresolved. Together, these results support context-dependent alteration in Polycomb-associated regulation, while the functional consequence of BCOR-ITD in human tumors remains unresolved. Retained PRC1.1 association also does not establish whether the complex occupies the same genomic sites.

**Figure 3:**
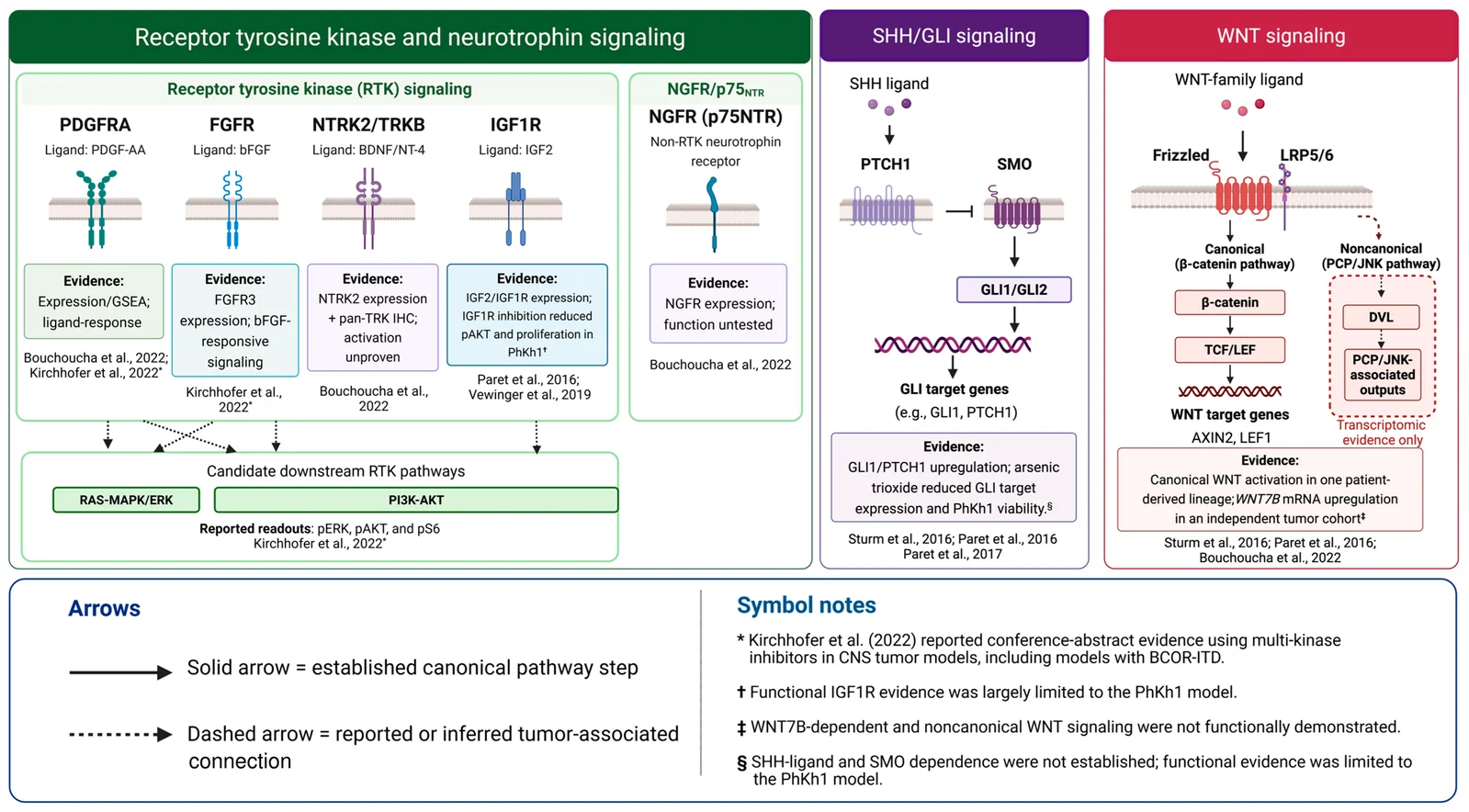
Candidate signaling pathways implicated in CNS tumors with BCOR-ITD [16,17,108,109,151,152]. Created in BioRender.com.

For *BCOR::CCNB3*, overexpression increased the proportion of NIH3T3 cells in S phase [14]. Tumor RNA profiling identified altered gene-expression profiles [85], while a single-case multi-omics study identified both gene expression and DNA methylation changes [128]. These transcriptional abnormalities do not by themselves establish that the fusion causes a differentiation block. These findings support fusion-associated effects but do not demonstrate recruitment of PRC1.1 to new loci. Other BCOR fusions retain different interaction regions and should be evaluated separately [83,130].

Because *BCOR* is located on the X chromosome, a dominant-negative effect on wild-type BCOR is unlikely to be the primary pathogenic mechanism in *BCOR*-altered tumors. If mutant BCOR is the only form expressed in a tumor cell, there is no wild-type BCOR protein present for it to inhibit or displace. In female CCSKs, both wild-type and ITD alleles were detected in tumor DNA, but mutant RNA was predominantly expressed [135]. Roy et al. estimated that mutant transcripts accounted for 96–100% of BCOR expression in the ITD-positive CCSKs analyzed, although wild-type RNA was also detected in female samples [94]. Because these measurements were obtained from bulk tumor samples, they cannot establish whether both proteins are expressed in the same tumor cells. Therefore, these data do not fulfill the conditions necessary to confirm a dominant-negative effect on wild-type BCOR. Whether CNS tumors show a similar pattern of allele expression remains unclear.

Experimental models also require careful interpretation. Introducing mutant BCOR into cells that retain endogenous wild-type BCOR creates a setting in which both proteins coexist [110]. Therefore, demonstrating a dominant-negative effect under this artificial condition would not, by itself, prove the same mechanism in primary tumors. Alternatively, mutant BCOR might inhibit wild-type BCORL1, or mutant BCORL1 might inhibit wild-type BCOR. Although BCOR and BCORL1 form separate PRC1.1 complexes in myeloid models [37], this finding does not establish whether either mutant protein directly inhibits the other protein’s function. Competition for shared PRC1.1 components is one possible mechanism that warrants further investigation. Validating this model would require clear evidence that the mutant protein impairs the function of complexes containing its wild-type counterpart, beyond the loss of its own normal activity. Table 3 summarizes the effects of different BCOR alterations on PRC1.1 function, chromatin regulation, and differentiation, together with the limitations of the available evidence.

**Table 3:** Mechanistic differences across major *BCOR* alteration classes. Abbreviations: aa, amino acid; ESC, embryonic stem cell; H2AK119ub1, monoubiquitination of histone H2A at lysine 119; ITD, internal tandem duplication; PRC1.1, Polycomb repressive complex 1.1; PRC2, Polycomb repressive complex 2; PUFD, PCGF ubiquitin-like fold discriminator.

Alteration Class	PRC1.1 and Chromatin Effects	Differentiation/Cellular Phenotype	References
*BCOR* loss-of-function (PUFD-disrupting truncation/loss)	Can uncouple PCGF1-RING from KDM2B targeting components; Reduced repression at tested loci; BCORL1-containing PRC1.1 complexes may retain residual activity after BCOR loss	Myeloid progenitor expansion and altered differentiation; Lineage/self-renewal gene derepression; Cooperation with additional oncogenic lesions in selected models	[37,59,60,61,107]
*BCOR::CCNB3* and other fusions	Altered gene-expression profiles; DNA-methylation changes reported in a single-case multiomics study; PRC1.1 retargeting unproven; Other fusions retain different BCOR regions and require separate evaluation	Increased S-phase fraction in NIH3T3 cells; Transcriptional abnormalities; Causal differentiation block unproven	[14,83,85,128,130]
*BCOR*-ITD (PUFD-region duplication)	30-aa PUFD duplication hinders PCGF1 binding/assembly in fragment assays; Full-length/endogenous ITD retains PRC1.1 association in mouse ESCs; H2AK119ub1 effects vary by model/construct	Developmental/PRC2-target-gene derepression in neural models; Abnormal differentiation under EZH2 inhibition in mouse ESCs. BCOR-ITD may largely retain wild-type function in ESCs while increasing susceptibility to Polycomb-pathway perturbation; Neomorphism, hypermorphism, and wild-type BCOR displacement unresolved	[45,110,111]

## 7. Therapeutic Vulnerabilities and Targeting BCOR Pathways

Potential therapeutic approaches vary according to *BCOR* alteration type and cellular context. Importantly, candidate pathways identified in experimental models should be distinguished from clinically validated therapeutic vulnerabilities. As summarized in Table 4, most reported vulnerabilities are supported by preclinical studies, retrospective associations, or limited clinical observations rather than validated clinical evidence. Further clinical studies are needed to determine whether *BCOR* status can guide treatment selection.

### 7.1. Drug Responses Associated with BCOR Loss of Function

In *BCR::ABL1*-positive K562 cells, *BCOR* loss, combined *BCOR*/*BCORL1* loss, and *KDM2B* loss increased *FGFR1* expression and promoted imatinib resistance [37]. In *BCOR*/*BCORL1* double-knockout cells, adding the FGFR inhibitor PD173074 to imatinib reversed this resistance. PD173074 alone did not suppress cell growth under the tested conditions. These findings suggest that the combination may help overcome imatinib resistance in this cell model.

In retinoblastoma cell models, experimental BCOR depletion increased IGF1-IGF1R signaling and sensitivity to the IGF1R inhibitors linsitinib and AEW541 [104]. Linsitinib also reduced tumor growth in BCOR-deficient flank and orthotopic xenografts. These findings remain preclinical. Further studies are needed to confirm these responses in retinoblastomas with naturally occurring *BCOR* mutations. Additional candidate approaches for MDS and AML are summarized in Table 4.

### 7.2. Candidate Therapeutic Approaches for BCOR-ITD Tumors

In the patient-derived PhKh1 culture, arsenic trioxide (ATO) reduced cell viability and GLI-target-gene expression, including *GLI1*, *GLI2*, and *PTCH1*, while the SMO inhibitors vismodegib and itraconazole showed limited activity [152]. Adding ATO to irradiation reduced colony formation compared with irradiation alone. However, these findings fell short of proving GLI dependency. One patient treated with ATO, surgery, chemotherapy, and reirradiation experienced a 6-month remission before systemic progression. Because the patient received several treatments, the contribution of ATO to this response remains unclear.

**Table 4:** Candidate therapies and resistance settings in BCOR biology. Abbreviations: AML, acute myeloid leukemia; ATO, arsenic trioxide; CNS, central nervous system; DHODH, dihydroorotate dehydrogenase; DLBCL, diffuse large B-cell lymphoma; DNMT, DNA methyltransferase; FGFR, fibroblast growth factor receptor; GC, germinal center; HDAC, histone deacetylase; IGF1R, insulin-like growth factor 1 receptor; ITD, internal tandem duplication; MDS, myelodysplastic syndrome; PDGFR, platelet-derived growth factor receptor; PRC1.1, Polycomb repressive complex 1.1; TKI, tyrosine kinase inhibitor.

Alteration and Setting	Strategy or Response	Evidence and Scope	Interpretation	References
*BCOR*/*BCORL1* loss in *BCR::ABL1* leukemia	Imatinib plus FGFR inhibition	Engineered K562 competition assays	Reversal of imatinib resistance in the tested model; broad TKI hypersensitivity is not established	[37]
*BCOR*-mutant MDS	Decitabine (hypomethylating therapy)	Retrospective decitabine-treated cohort: complete remission in 8/14 (57.1%) *BCOR*-mutant versus 52/181 (28.7%) *BCOR*-wild-type patients.	Clinical association with response; not prospective validation or proof of BCOR-specific drug sensitivity	[153]
*BCOR*-mutant/deficient AML	DHODH inhibition (brequinar; leflunomide)	Multiple cellular models using genetic and chemical *BCOR* perturbation	Preclinical synthetic vulnerability; no established clinical efficacy in *BCOR*-mutant AML	[154]
BCOR-associated HDAC/DNMT repression	HDAC-directed or combined epigenetic strategies	BCOR-HDAC interaction and context-specific DNMT recruitment studies; no *BCOR*-selective treatment-response data	Mechanistic rationale only; therapeutic sensitivity remains unproven	[1,22]
Experimental *BCOR* loss in retinoblastoma	IGF1R inhibition	Cell assays; subcutaneous and orthotopic xenografts (typically 5–7 mice/group)	Preclinical efficacy; validation in patient-derived *BCOR*-mutant models is needed	[104]
CNS tumor with BCOR-ITD	ATO with irradiation	PhKh1 culture; one patient treated with a multimodal relapse regimen	Clinical response cannot be attributed to ATO alone	[108,152]
CNS tumor with BCOR-ITD	IGF1R blockade or ceritinib	Single patient-derived functional culture (PhKh1); expression data from three tumors	Preclinical, single-model evidence; no established clinical efficacy	[109]
CNS tumors with varied *BCOR*/*BCORL1* alterations	FGFR/PDGFR-active multikinase inhibitors	Conference abstract: four models from three patients (two ITD models from one patient); two patients received multimodal regimens including nintedanib	Conference-abstract evidence only; receptor/allele specificity and clinical efficacy remain unvalidated	[151]
*BCOR*-rearranged uterine sarcoma	Candidate CDK4 or MDM2 targeting	Genomic co-alterations	Genomic rationale only; drug dependency untested	[89,90,93]
Intact *BCOR* in GC-derived DLBCL	Dual EZH2/BCL6 inhibition	Cell lines; two xenograft models; four primary specimens ex vivo	Mechanistically supported preclinical strategy; BCOR mutation status is not an established response biomarker	[64]
*BCOR* loss or PRC1.1 disruption in *KMT2A*-rearranged AML	Menin-inhibitor resistance	Cell perturbations; PCGF1-deficient xenograft resistance	Candidate resistance biomarker, not a therapeutic vulnerability	[119,120]

IGF1R inhibition and ceritinib treatment also reduced PhKh1 cell viability [109]. Ceritinib additionally reduced IGF2-stimulated phosphorylation of IGF1R and AKT. However, these findings were obtained from the same patient-derived culture. Further studies in independent models are needed to determine whether other CNS tumors with BCOR-ITD respond similarly.

### 7.3. Candidate Therapies for BCOR and BCORL1 Fusions and Other Alterations

A conference abstract reported that multikinase inhibitors targeting FGFR and PDGFR reduced viability and MAPK and PI3K/AKT signaling across four CNS cell models derived from three patients [151]. These included two *BCOR*-ITD models from the same patient. Two patients received nintedanib alongside other treatments. Complete remission was reported in a patient with a *BCOR* frameshift mutation and a *BCORL1* fusion, while a patient with *BCOR*-ITD showed disease stabilization. Because these drugs inhibit several kinases, the responses do not establish dependence on a specific receptor. Furthermore, concomitant therapies obscure the precise clinical contribution of nintedanib.

Studies of endometrial stromal sarcomas with *BCOR* or *BCORL1* alterations have identified *CDK4* and *MDM2* amplification and *CDKN2A* deletion in a subset of tumors, suggesting possible treatment approaches [89,90,93]. Further experiments are needed to determine whether these changes increase sensitivity to targeted treatments and whether such sensitivity depends on the underlying *BCOR* or *BCORL1* alteration.

### 7.4. Targeting BCOR-Associated Repression in GCB-DLBCL

Combined EZH2 and BCL6 inhibition showed greater antitumor activity than either inhibitor alone in germinal center B-cell-type (GCB) DLBCL cell models [64]. Similar findings were reported in two xenograft models and four primary tumor samples tested ex vivo. This approach is based on cooperation between BCL6 and EZH2 in recruiting and stabilizing a specialized CBX8-containing BCOR repressive complex at bivalent promoters. These findings support targeting this repressive mechanism, but do not establish *BCOR* loss-of-function status as a marker for patient stratification.

### 7.5. PRC1.1 Disruption as a Candidate Biomarker of Treatment Resistance

Disruption of PRC1.1 components, including BCOR, reduced sensitivity to menin inhibitors in *KMT2A*-rearranged AML cell models [119]. Menin-inhibitor resistance was also observed in mice transplanted with *PCGF1*-deficient leukemia cells. A later study showed that *BCOR* or *PCGF1* loss reduced sensitivity to menin and DOT1L inhibitors and weakened repression of leukemia-associated genes after treatment [120]. These findings suggest that PRC1.1 disruption may be a marker of treatment resistance. However, further clinical studies are needed to determine whether specific *BCOR* variants predict treatment response.

## 8. Conclusions and Future Perspectives

Although *BCOR* mutations are now well-recognized in developmental disorders and diverse malignancies, a major challenge remains in determining how individual alterations dictate distinct molecular and cellular phenotypes. In many contexts, it is still unclear how a specific mutation impacts BCOR protein stability, physical interactions with its binding partners, chromatin recruitment, and downstream transcriptional regulation. Consequently, functional insights gleaned from a single *BCOR* mutation may not generalize to others. Future studies must systematically delineate how loss-of-function mutations, gene fusions, and ITDs alter BCOR function within the specific cellular and developmental contexts. Experimental models that preserve physiological expression levels and control for genetic background will be paramount in identifying phenotypic effects directly attributable to each unique genetic alteration.

Linking these molecular consequences to shifts in cell identity, differentiation, and proliferation will be critical to deciphering how each alteration drives disease progression. Longitudinal assessments of molecular changes across different chronological stages may further distinguish primary oncogenic drivers from secondary compensatory mechanisms that emerge during differentiation or cell-state transitions. Additionally, further investigation into BCORL1 is required to clarify its functional relationship with BCOR and explain why alterations in these two genes are associated with different developmental and cancer phenotypes.

At present, *BCOR* mutations possess greater clinical value for molecular diagnosis and tumor classification than for guiding therapeutic interventions. Although several signaling and epigenetic vulnerabilities have been reported in malignancies with *BCOR* alterations, most findings are currently constrained by small patient cohorts and limited experimental models. Consequently, the reproducibility and generalizability of these targetable dependencies across broader patient populations remain unverified. Overcoming these barriers will require larger patient cohorts, diverse disease-relevant models, and integrated multiomic and clinical analyses to elucidate the precise pathogenic mechanisms and identify robust therapeutic targets. Ultimately, these efforts will be essential to support the development of more reliable biomarkers and effective, personalized treatment strategies.

## Data Availability

No new data were created or analyzed in this study. Data sharing is not applicable to this article.
